# Development of cereal‐based functional food using cereal‐mix substrate fermented with probiotic strain – *Pichia kudriavzevii* OG32

**DOI:** 10.1002/fsn3.239

**Published:** 2015-04-30

**Authors:** Omotade R. Ogunremi, Renu Agrawal, Abiodun I. Sanni

**Affiliations:** ^1^ Department of MicrobiologyUniversity of IbadanIbadanNigeria; ^2^ Food Microbiology DepartmentCentral Food Technological Research InstituteMysoreIndia

**Keywords:** Cereal, fermentation, *Pichia kudriavzevii *OG32, probiotics, volatile compounds

## Abstract

Probiotic strains contribute to the functionality of foods during fermentation. In this present work, cereal‐mix was fermented with probiotic *Pichia kudriavzevii* OG32. Selected fermentation parameters and functional properties of the product were determined. The growth of *Pichia kudriavzevii* OG32 was supported by the cereal‐mix containing 1% salt and 0.2% red chili powder to counts of between 7.46 and 8.22 Log_10_ cfu/mL within 24 h. Pichia kudriavzevii OG32 increased the viscosity of cereal‐mix with the highest inoculum size (1.84x105cfu/ml) giving the highest viscosity of 1793.6 mPa.S. An inoculum size of 1.98 × 10^4^ cfu/mL gave the most acceptable product based on the sensory evaluation by the panelist. Forty volatile compounds were identified in the fermented product, while acids (32.21%) and esters (32.37%) accounted for the largest proportions. The cereal‐based fermented product scavenged DPPH from 200 *μ*mol/L methanolic solution by 55.71%. Probiotic yeast improved the sensory and some functional properties of cereal‐based substrate during fermentation. This is one of the first reports on the volatile composition of cereal‐based functional food produced with probiotic yeast.

## Introduction

Functional foods are food products that provide health benefits beyond the traditional nutrients (Williams et al. 2006). Increased awareness of consumer health, cost of health care and steady increase in life expectancy have resulted in the demand for food products that can improve consumer well‐being (Lamsal and Faubion 2009; Gupta and Abu‐Ghannam 2012). Probiotic strains contribute to the functional properties of food during fermentation (Jespersen 2003). Although, dairy products are the prominent probiotics, nondairy products, especially cereals are becoming the favored choices as probiotics delivery vehicles (Prado et al. 2008; Lamsal and Faubion 2009; Granato et al. 2010).

Cereals are very important crops used for human nutrition in most parts of the world. They are readily available because they are grown in greater quantities where they constitute important sources of nutrients, phytochemicals, and other bioactive compounds. Cereal grains are very good substrates for fermentation globally and the predominant microorganisms are lactic acid, bacteria, and yeasts (Blandino et al. 2003; Franz et al. 2014).

The choice of cereal‐based substrate for the development of probiotic foods is motivated by increase in consumer vegetarianism, lactose intolerance, cholesterol content, and economic reasons that are associated with dairy products (Prado et al. 2008; Gobbetti et al. 2010). Cereals also have the potentials to offer consumer prebiotic and whole grain benefits (Lamsal and Faubion 2009). There are few reports on the suitability of cereals as carrier of probiotic lactic acid bacteria but no work has reported the use of probiotic yeast strains to develop cereal‐based probiotic food despite reports supporting the probiotic potentials of yeasts and the wide physiological diversity of yeasts in traditional fermented cereal‐based foods.

Probiotic yeast is required to be technologically suitable to influence the functionality of cereal substrate during fermentation. The strains must be able to attain high population (10^6^–10^8 ^g/mL), retain desired properties, and provide desirable organoleptic effects when included in cereal fermentation processes (Klaenhammer and Kullen 1999; Gupta and Abu‐Ghannam 2012).

There are several features of probiotic yeasts that can contribute to their successful inclusion during fermentation to produce cereal‐based functional foods. Yeasts maintain viability and stability over changing food processing conditions. They can tolerate a wide range of salt concentration, oxygen activity, and pH (Arroyo‐López et al. 2008; Walker 2009; Nayak 2011; Bonatsou et al. 2015). Yeasts impart desirable organoleptic properties on fermented foods. Choice yeast species, during cereal fermentation metabolize sugars to a wide range of flavor‐active compounds, constituting the distinctive flavor of the product (Combina et al. 2005; Tamang and Fleet 2009).

In this study, we developed a cereal‐based functional food using cereal‐mix made up of sorghum, pearl millet, and wheat with probiotic *Pichia kudriavzevii* OG32. The aim of the study was to investigate the influence of the probiotic yeast strain on some organoleptic and functional properties of the cereal‐based substrate.

## Materials and Methods

### Probiotic starter culture

Probiotic strain – *Pichia kudriavzevii* OG32 was used in this study. The strain was isolated from *ogi* (fermented cereal gruel) and selected as the probiotic based on in vitro studies. The strain was maintained on Yeast Peptone Dextrose (YPD) agar (Himedia, Mumbai, India) at 5°C. Starter culture was obtained after overnight incubation at 37°C in YPD broth. The culture was centrifuged at 7000 rev/min and 4°C for 10 min, pellets were washed three times in 0.1% sterile NaCl and resuspended in 0.1% sterile NaCl to its original volume (Angelov et al. 2006). The cell count was standardized to 10^7 ^cfu/mL and serial dilution was carried out to obtain 10^6^ and 10^5 ^cfu/mL.

### Cereal substrates and slurry preparation

**Figure 1:**
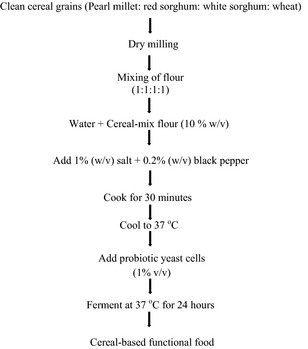
Flow chart for the preparation of cereal‐based functional food.

The whole grains of clean cereals, including, white and red sorghum (*Sorghum bicolor*), pearl millet (*Pennisetum glaucum*), and wheat (*Triticum aestivum*) were dry‐milled separately using a Christy Briton hammer mill (Model LB7, Christy‐Turner, Suffolk, UK). Slurry was prepared by mixing equal proportions of each of the flour (1:1:1:1) in distilled water (10% w/v). According to a modified method of Angelov et al. (2006), different combination of sweeteners and spices were added to equal volumes of the slurry as follows: (1) 1.5% sugar, (2) 3% sugar + 0.1% cardamom powder, (3) 1% salt, (4) 1% salt + 0.2% black pepper powder, (5) 1% salt + 0.2% red chili powder, and (6) 1% salt + 0.1% red chili powder + 0.1% black pepper powder. Sweeteners and spices were sourced from local markets in Mysore, India. The starch in the slurry was gelatinized and hydrolyzed by stirring the suspension at 95°C for 30 min, and then allowed to cool to 37°C. Sensory evaluation was performed to determine the most acceptable combination of sweeteners and spices before fermentation studies. The sensory panel was made up of 10 members that rated the samples for physical appearance, consistency, taste, and overall quality on a nine‐point Hedonic scale with nine representing “like extremely” and 1 represent “dislike extremely”. A flow chart of the slurry preparation and fermentation is shown in Figure 1.

### Fermentation

Two hundred and fifty milliliters of sterile slurry was inoculated with 1% (v/v) of the different concentrations of the yeast starter culture (10^7^, 10^6^ and 10^5^_ _cfu/mL) to give a final cell concentration of 10^5^, 10^4^, and 10^3^_ _cfu/mL in the respective fermenting matrix. Uninoculated slurry served as control. Fermentation was carried out at 37°C for 24 h and samples were aseptically taken at the end of the fermentation for further analysis (Angelov et al. 2006; Rathore et al. 2012).

### Enumeration of viable cells and determination of pH

Enumeration of viable yeast cells was performed by estimating the colony‐forming units (cfu/mL) from the samples using YPD agar at 30°C for 24 h. The pH of the samples was determined with the use of a pH meter (GENEI, Bangalore, India).

### Determination of viscosity

The viscosity of 100 mL of each sample was determined using a viscometer (Model: R1:3: M, Rheology International Shannon Ltd., Shannon, County Clare, Ireland) equipped with spindle no. 6 rotating at 50 rev/min at 25°C for 30 sec. Viscosity was expressed in milliPascal second (mPa sec) (Vijayendra and Sharath Babu 2008).

### Sensory evaluation

The most acceptable product after fermentation was determined by a sensory panel of 10 members. They rated the samples for appearance, aroma, taste, and overall acceptability by using a nine‐point Hedonic scale (9 and 1 representing “like extremely” and “dislike extremely” respectively).

### Determination of volatile compounds

A 10 mL sample of the most acceptable fermented product based on the sensory evaluation was taken to determine the volatile compounds.

#### Volatile compounds extraction

An equal volume of dichloromethane (SD Fine Chemicals, Mumbai, India) was added to the sample, poured into a 100 mL separatory funnel and shaken vigorously for 2 min. It was allowed to separate until the solvent and aqueous phases were formed. The solvent phase (dichloromethane) was gently released into a dry test tube. The procedure was repeated twice and the solvent phases were pooled into the test tube and 0.5 g of anhydrous sodium sulphate was added. The test tube was kept for 5 min for the total removal of moisture before transferring the extract into a graduated tube. The extract was concentrated to 500 *μ*L by shaking in a water bath at ambient temperature (Vanaja et al. 2011).

#### Analysis of volatile compounds

The concentrated extract was analyzed for volatile compounds using gas chromatography‐mass spectrometer (GC‐MS) (PerkinElmer‐Autosystem XL/TurboMass Gold, Waltham, Massachusetts, USA). The instrument is equipped with an ELITE 1 nonpolar capillary column (30 m × 0.25 mm (ID); 0.25 *μ*m film thickness). Separation of volatiles in 1 *μ*L (split ratio 1:10) of the extract was carried out along the capillary column by helium gas at a flow rate of 1 mL/min. The oven temperature was held at 100°C for 6 min, heated at 4°C/min to 150°C, then 8°C/min to 220°C, and held at 220°C until an approximate run time of 40 min. The mass spectrophotometer was operated in the electron impact mode and mass spectra were taken, using an ionization voltage of 70 eV. The mass scan range was 40–400 AMU, with a scanning speed of 0.2 sec. Data acquisition and generation of chromatograms and mass spectra for each peak was done with PerkinElmer software (TurboMass 5.4 GC/MS) (Vanaja et al. 2011).

#### Identification and quantification of volatile compounds

The identification of volatile compounds was performed by comparing the mass spectra with standard mass spectra database from the NIST Ver. 2.1 2009 Mass Spectra Library, Gaithersburg, Maryland, USA. The relative percentage of each volatile compound was calculated by comparing the peak area with the total area as follows.

$$\begin{array}{c} \text{Relative percentage of each volatile compound}\;=\\ \;\frac{\text{Compound peak area}}{\text{Total compounds peak area}}\;\times \;100 \end{array}$$

### Determination of antioxidant activity

A 5 mL sample of the fermented product was taken and extracted for antioxidant assay. Extraction was carried out in 25 mL of deionized water: methanol (1:1) using a modified method of Noipa et al. (2011). The mixture was homogenized in mortar and pestle, at 5°C for 15 min. The solution was centrifuged at 7500 rev/min for 10 min at 4°C, and the supernatant was collected. One milliliter of the supernatant was used for the determination of antioxidant activity by using the 1,1‐diphenyl‐2‐picryl‐hydrazyl (DPPH*) free radical scavenging method (Sharma and Bhat 2009).

$$\text{Antioxidant activity}\;\left(\%\right)\;=\;\frac{A0-A}{A0}\;\times \;100$$

*A0*: Optical density of control, *A*: Optical density of sample extract.

### Statistical analysis

Results represented means with standard deviation of triplicate values. Data obtained were subjected to ANOVA and the significant differences were compared using Duncan's Multiple Range Test (DMRT). Values of *P* < 0.05 were considered statistically significant.

## Results

### Slurry preparation

Results of sensory evaluation of the cereal‐mix after the addition of different combinations of sweeteners and spices showed that the addition of 1% salt and 0.2% red chili powder provided the most acceptable overall quality (Table 1). This combination of additives was selected for the fermentation studies. The different combinations of sweeteners and spices had no effect on the consistency but they significantly affected the physical appearance and taste of the cereal mix. The cereal mix slurry to which 1% salt, 0.1% red chili powder, and 0.1% black chili powder were added had the best physical appearance while the addition of 1.5% sugar gave the least acceptable taste (Table 1).

**Table 1:** Sensory evaluation of cereal‐based slurry prepared with different combinations of sweeteners and spices

Sample	Appearance	Consistency	Taste	Overall quality
A	5.40 ± 0.55^a^	5.85 ± 0.02^a^	3.60 ± 0.10^a^	4.95 ± 0.84^a^
B	5.40 ± 0.34^a^	5.85 ± 0.06^a^	4.95 ± 0.07^b^	4.95 ± 0.82^a^
C	5.40 ± 0.77^a^	5.85 ± 0.02^a^	5.06 ± 0.31^bc^	5.40 ± 1.11^b^
D	6.30 ± 0.58^b^	5.85 ± 0.03^a^	6.75 ± 0.11^e^	5.85 ± 1.02^c^
E	6.30 ± 0.23^b^	5.85 ± 0.03^a^	5.40 ± 0.21^c^	5.06 ± 1.22^ab^
F	6.30 ± 0.72^b^	5.85 ± 0.04^a^	6.30 ± 0.03^d^	5.85 ± 0.96^c^

Data are means with standard deviations of triplicate values. Values not sharing a common superscript differ significantly at *P *<* *0.05 (DMRT).

Key: 1.5% sugar (A), 3% sugar + 0.1% cardamom powder (B), 1% salt (C), 1% salt + 0.2% black pepper powder (D), 1% salt + 0.2% red chili powder (E), 1% salt + 0.1% red chili powder + 0.1% black pepper powder (F).

### Viable cells count and pH

The addition of 1% (v/v) of different cell concentrations of probiotic *Pichia kudriavzevii* OG32 to the cereal mix slurry achieved cell counts of 10^3^, 10^4^, and 10^5 ^cfu/mL at the onset of fermentation. The yeast counts obtained after 24 h of fermentation are presented in Table 2. Colony counts from the range of 10^6^–10^7^ cfu/mL were achieved for all the samples. This is an indication that *Pichia kudriavzevii* OG32 grew and retained viability in the cereal mix slurry. Cell concentrations within this range are required to provide the desired probiotic effects. Reduction in pH was recorded from pH 6.01 to pH 5.08 at the end of fermentation (Table 2).

**Table 2:** pH and viable count during laboratory fermentation of cereal‐based slurry using different inoculum sizes of *Pichia kudriavzevii* OG32 as starter cultures

Sample	pH		Viable count (Log_10_ cfu*/*mL)	
	Initial	Final	Initial	Final
Control	6.01 ± 0.01	5.98 ± 0.01	–	–
A	6.01 ± 0.01	5.14 ± 0.08	4.37 ± 0.21	7.46 ± 0.22
B	6.01 ± 0.01	5.10 ± 0.05	5.30 ± 0.15	8.19 ± 0.14
C	6.01 ± 0.01	5.08 ± 0.09	6.27 ± 0.62	8.22 ± 0.31

Data are means with standard deviations of triplicate values.

### Viscosity

**Figure 2:**
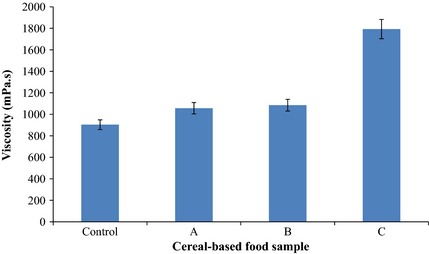
Visosity of cereal‐based functional food prepared using different inoculum sizes of *Pichia kudriavzevii* OG32. Data are means with standard deviations of triplicate values. Key: Control: No inoculum; A: Inoculum size (2.3 × 10^3^ cfu/mL); B: Inoculum size (1.98 × 10^4^ cfu/mL); C: Inoculum size (1.84 × 10^5^ cfu/mL).

There was a gradual increase in the viscosity of the fermented products as the inculum size increased (Fig. 2). Sample D with inoculum size of 10^7 ^cfu/mL had the highest viscosity (1793.6 mPa.s).

### Sensory evaluation

Result from sensory evaluation of the fermented product, using different inoculum sizes of *Pichia kudriavzevii* OG32 is as presented in Table 3. There was no significant difference in the observed consistency between the control sample and the fermented products using different inoculum concentrations. Inoculum size of 10^4^ cfu/mL was found to provide the most acceptable taste and flavor. There was a gradual increase in appealing mouth‐feel with increasing inoculum concentration. Sample C, with inoculum concentration of 10^6 ^cfu/mL was the most acceptable fermented product.

**Table 3:** Sensory evaluation of cereal‐based functional food prepared with different inoculum sizes of *Pichia kudriavzevii* OG32

Sample	Appearance	Consistency	Taste	Mouth feel	Overall quality
Control	5.85 ± 0.14^a^	6.75 ± 0.03^a^	4.95 ± 0.09^ab^	4.05 ± 0.12^d^	5.40 ± 0.21^a^
A	5.40 ± 0.67^ab^	6.75 ± 0.09^a^	3.60 ± 0.16^b^	4.50 ± 0.31^c^	4.50 ± 0.25^b^
B	5.40 ± 0.42^ab^	6.75 ± 0.03^a^	5.85 ± 0.77^a^	4.95 ± 0.22^b^	5.85 ± 0.46^a^
C	4.95 ± 0.33^b^	4.05 ± 0.11^ab^	3.60 ± 0.30^b^	5.85 ± 0.08^a^	4.05 ± 0.16^b^

Data are means with standard deviations of triplicate values. Values not sharing a common superscript differ significantly at *P *<* *0.05 (DMRT).

Control: No inoculum; A: Inoculum size (2.3 × 10^3^); B: Inoculum size (1.98 × 10^4^); C: Inoculum size (1.84 × 10^5^).

### Volatile compounds

**Figure 3:**
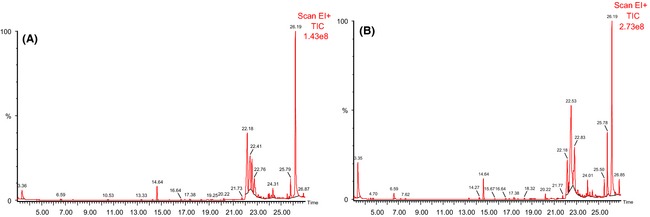
Gas chromatograms of the volatile compounds produced by *Pichia kudriavzevii* OG32 in cereal‐based functional food: (A) Control; (B) Fermented product. Peak identities are shown in Table 4.

Gas chromatograms of the volatile compounds in the fermented cereal‐based product and control sample are shown in Figure 3. The number and relative percentages of volatile compounds are shown in Table 4. *Pichia kudriavzevii* OG32 enhanced the formation of volatile flavor compounds in the fermented product (Table 4). A total of 44 volatile compounds were identified in this study. They were broadly categorized into six groups, including acids (9), alcohols (11), alkanes (6), aromatics (3), carbonyls (8), and esters (7). Forty (40) of the volatile compounds were identified in the fermented product sample and eighteen (18) were identified in the control sample. Several volatile compounds occurred at high concentrations in both the fermented cereal‐based product and control sample. Acids (32.21%) and esters (32.37%) had the largest proportion of the volatile compounds in the fermented cereal‐based product while alkanes and aromatic compounds had the least proportion (6.02%). Acids accounted for 68.4% of the volatile compounds detected in the control food. The major acids produced in both samples were 9, 12‐octadecadienoic acid, and acetic acid (Table 4). Benzyl alcohol and 2‐Hexyl‐1‐octanol were found to be the predominant alcohols in both samples (Table 4). 9‐Octadecenal is the only carbonyl that was common to both samples. 9, 12‐Octadecadienoic acid methyl ester was the predominant ester in the control (6.87%) and fermented product (25.02%) (Table 4).

**Table 4:** Volatile compounds of cereal‐based functional food prepared with *Pichia kudriavzevii* OG32

Retention time	Compound	Control (%)	Cereal‐based functional food (%)
3.2	5,7‐Dimethyl Undecane	–	0.04
3.4	Benzyl alcohol	2.62	6.63
4.5	Nonanal	–	0.13
4.7	Dimethyl acetal	–	0.13
7.2	Undecanal	–	0.04
7.6	3‐Methyl, 4‐Heptanone	–	0.09
13.3	Alpha‐cubebene	–	0.10
14.3	4,6‐Dimethyl Undecane	–	0.14
14.6	Caryophyllene	2.30	2.18
15.7	Dextro‐linalyl acetate	–	0.05
17.4	Geranyl acetate	–	0.23
18.3	Dihydrocarvy acetate	–	0.05
18.8	Dodecatrien‐3‐ol	0.44	–
18.9	Tetradecanoic acid	–	0.04
21.4	Tetramethyl decane	–	0.20
21.6	Propanoic acid	–	0.04
21.7	10‐Undecen‐1‐ol	0.44	–
21.8	Pentdecen‐1‐ol	–	0.54
22.2	9,12‐Octadecadienoic acid	18.54	4.43
22.3	2‐Hexy‐1‐octanol	11.89	1.21
22.5	9.12‐Octadecadienoic acid methyl ester	6.87	25.02
22.6	9‐Hexadecenoic acid	6.88	–
22.8	11‐Tetradecenoic acid	5.05	–
22.9	13‐Octadecenal	–	9.56
23.0	9‐Tetradecenoic acid	0.47	0.02
23.1	3‐Pentadecen‐2‐ol	–	0.77
23.2	Tetradecanal	–	0.17
23.3	Octadecanal	–	0.35
23.4	Dodecanoic acid 2‐penten‐1‐yl ester	–	0.04
23.5	18‐Nonadecen‐1‐ol	–	0.21
23.7	11,14‐Eicosadienoic acid methyl ester	–	0.19
23.9	9‐Octadecenal	0.87	0.49
24.0	Octadecane	0.78	0.91
24.1	9‐Heptadecanol	0.77	0.06
24.3	12‐methyl, teradecanoic acid	1.49	0.07
24.4	1,2 Benzendicarboxylic acid	0.61	0.63
24.7	6,10‐Dimethyl‐4‐undecanol	–	0.41
24.8	2‐Hexyl‐1‐Decanol	–	0.11
24.9	1‐Tetracosanol	–	0.12
25.5	Nonadecane	0.74	1.77
25.8	Dibuty phthalate	2.92	6.79
26.2	Acetic acid	35.36	27.98
26.5	2(3H)‐Furanone	–	0.68
26.9	Tetramethyl heptadecane	0.54	–

–: Not detectable.

Percentage proportion of volatile compound is expressed as (compound peak area/total compounds peak area) × 100. Data are means of triplicate values.

### Antioxidant activity

**Figure 4:**
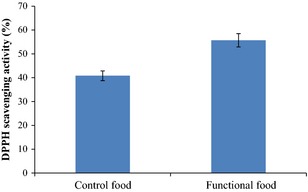
Antioxidant capacity of cereal‐based functional food prepared with *Pichia kudriavzevii* OG32. Data are means with standard deviations of triplicate values.

There was an increase in the antioxidant activity of fermented product upon the incorporation of the probiotic *Pichia kudriavzevii* OG32 based on the result shown in Figure 4. The fermented product scavenged DPPH* from 200 *μ*mol/L methanolic solution by 55.71%, while the control sample recorded a scavenging capacity of 40.83%.

## Discussion

Probiotic microorganisms participate in the fermentation of various foods, generating bioactive components that enhance the functionality of food products (Yadav et al. 2011). These foods also serve as delivery vehicles of probiotic strains to the desired target sites in the human or animal body. To achieve health benefits, functional foods are expected to support the growth and maintain strains' viable count in the range of about 10^6^–10^7^ cfu/g of the product (Lamsal and Faubion 2009). Naturally fermented cereals account for up to 80% of total calorie consumption in many African countries (Franz et al. 2014). The increase in consumer vegetarianism, economic reasons, and whole grain benefits make cereals the favored choice for the delivery of probiotic strains (Prado et al. 2008). In this study, *Pichia kudriavzevii* OG32 was selected for the development of a cereal‐based functional food due to its important technological and functional properties. The cereal‐based food in this study was prepared from a mix of equal proportions of white sorghum, red sorghum, millet, and wheat after determination of the best sweetener and spice combination in a sensory evaluation. The sensory panel suggested that the addition of 1% salt and 0.2% red chili powder provide the most acceptable overall quality. It was observed that salt and red chili powder did not affect the viability of *Pichia kudriavzevii* OG32. This is in agreement with a report by Angelov et al. (2006), whereby the addition of sweeteners; aspartame, sodium cyclamate, saccharine and Huxol did not affect the viability of probiotic *Lactobacillus plantarum* B28 during the fermentation of oat mash. Sensitivities vary among strains, however, incorporated probiotic strains are required to maintain viability despite the addition of ingredients or additives during food processing (Champagne et al. 2005). Yeasts have been reported to grow at various stress conditions, including high salt concentrations (Lopandic et al. 2006; Walker 2009; Bonatsou et al. 2015).

The complement of the cereals used in this study is expected to provide the nutritional requirements for yeast growth. According to FAO (1999), each of the cereals that constitute the cereal mix in this study has a significant amount of vitamins and minerals. Pearl millet has the highest proportion of calcium and iron. In addition, wheat and pearl millet have higher protein content. Appropriate processing of cereals increases the amount of fermentable sugars for the growth of lactic acid bacteria and yeasts (FAO 1999; Charalampopoulos et al. 2002; Angelov et al. 2006). *Pichia kudriavzevii* OG32 showed good growths in our cereal mix slurry after 24 h. The strain gave viable counts above the suggested minimum limit (Log_10_ 6–7) required to deliver desired health benefits (Lamsal and Faubion 2009). Yeasts participate actively during food fermentation and they can achieve populations around 10^7^ cfu/mL in different food substrates (Fleet 1999). Our observation is consistent with reports using probiotic lactic acid bacteria as the starter culture in cereal fermentation. Sanni et al. (2013) reported viable cells counts in the range of 8.26–8.82 Log_10_ cfu*/*mL of probiotic Lactobacillus strains after fermenting sorghum slurry for sorghurt production. In addition, within 10 h, probiotic *L. plantarum* B28 showed growth by an increase of at least Log order of two in oat mash (Angelov et al. 2006). The viable cells count of *L. plantarum* increased above the suggested minimum limit of 6–7 Log_10_ cfu/mL after 6 h of fermenting single malt, barley, and barley malt (mixed cereal) (Rathore et al. 2012). The growth of *Pichia kudriavzevii* OG32 in the cereal mix slurry is attributed to the availability of fermentable sugars and other growth factors in the substrate (Charalampopoulos et al. 2002). There was an improvement in the mouth feel and viscosity of the food product as the inoculum size increased. This could be due to exopolysaccharides produced by the yeast strain and the increase in biomass density.

The flavor of food depends on the balance of volatile compounds that are inherently present in food or those produced during processing (Linton and Wright 1993). A vast number of volatile compounds are synthesized and modulated by yeasts during fermentation. They significantly impart the overall quality of the product (Callejon et al. 2010). These compounds include acids, higher alcohols, carbonyls, and esters. This study revealed the ability of *Pichia kudriavzevii* OG32 to enhance the flavor properties of cereal substrate. Forty (40) volatile compounds were identified in the fermented products while eighteen (18) were identified in the control sample. Previous studies have reported an increase of volatile compound due to fermentation by yeasts and lactic acid bacteria (Annan et al. 2003; Callejon et al. 2010; Parkouda et al. 2011; Sørensen et al. 2011). The most abundant volatile compounds in the functional food are acids and esters accounting for 32.21 and 32.37%, respectively. The available information in the literature affirmed the flavor properties of some of the detected volatile compounds. Esters mainly determine the pleasant aromatic notes of fermented products, contributing to the flora and fruity odors (Chen and Xu 2010; Moreira et al. 2011; Parkouda et al. 2011). Flavor‐active acids detected in the fermented product include tetradecanoic acid, which has a creamy and cheesy flavor and 9, 12‐octadecadienoic acid with a faint fatty flavor (The Good Scent Company (TGSC) 1989). These compounds were reported in unfermented and fermented baobab seeds (Parkouda et al. 2011). The proportion of higher alcohols was observed to be low in the fermented food (10.12%) and relatively low concentrations of higher alcohols contribute to fruity‐like aromas for foods while at higher concentrations they contribute to the ‘hot’ and ‘irritating’ aromas, which are undesirable to the consumer (Saberi et al. 2012). Benzyl alcohol is the largest proportion of alcohol in the fermented food. Benzyl alcohol has been reported in organic red wines fermented with *Saccharomyces cerevisiae* (Callejon et al. 2010). It has sweet, floral, and fruity flavor with balsamic nuances (The Good Scent Company (TGSC) 1989).

The total antioxidant activity of fermented food is attributed to the different antioxidants that are components of the food substrates and metabolites of the microorganisms. These antioxidants include enzymes such as catalase, glutathione reductase and superoxide dismutase; micro‐ and macromolecules such as ascorbic acid, folate, reduced glutathione, beta‐carotene, beta‐glucan, phenols, and fatty acids (Abbas 2006; Balasubramanian and Ragunathan 2012). A measure of the overall antioxidant capacity of the food may provide more relevant biological information compared to that obtained by the measurement of individual components. In this study, the overall antioxidant capacity of the cereal‐based food increased after fermentation by *Pichia kudriavzevii* OG32. A similar report by Qian et al. (2012) showed an increase in the antioxidant capacity of *Pavlova lutheri* (microalgae) after fermentation with *Hansenula polymorpha* (*Pichia angusta*). This could be due to yeasts' cell components or metabolites with antioxidant activity. Oxygenated carotenoids, organic acids, glutathione, some uncharacterized proteins and cell wall *β*‐glucan from yeasts have been characterized to have antioxidant properties (Abbas 2006; Balasubramanian and Ragunathan 2012).

## Conclusion

This study is able to suggest the suitability of cereal‐based matrix as a viable delivery vehicle for probiotic yeasts. The probiotic yeast grew above the minimum level required for probiotic effects. Inoculation with the probiotic yeast has an impact on the organoleptic and functional properties of the cereal‐mix slurry. In particular, there were increments the variety of flavor active compounds and antioxidant activity of the fermented product.

## Conflict of Interest

None declared.
